# Spatial distribution of gamma radiation levels in surface soils from Jaduguda uranium mineralization zone, Jharkhand, India, using γ-ray spectrometry, and determination of outdoor dose to the population

**DOI:** 10.4103/0971-6203.71762

**Published:** 2010

**Authors:** Mandakini Maharana, Narayani Krishnan, D. Sengupta

**Affiliations:** Department of Geology & Geophysics, Indian Institute of Technology, Kharagpur, West Bengal, India; 1 Radiation Safety Systems Division, Bhaba Atomic Research Center, Trombay, Mumbai, India

**Keywords:** Gamma dose, HPGe, Natural radioactivity, singhbhum shear zone, surface soil

## Abstract

The concentrations of natural radionuclides in surface soil samples around selected villages of Jaduguda were investigated and compared with the radioactivity level in the region. Concentrations of ^238^U, ^232^Th, and ^40^K were determined by a gamma ray spectrometer using the HPGe detector with 50% relative efficiency, and the radiation dose to the local population was estimated. The average estimated activity concentrations of ^238^U, ^232^Th, and ^40^K in the surface soil were 53.8, 44.2 and 464.2 Bq kg^−1^ respectively. The average absorbed dose rate in the study area was estimated to be 72.5 nGy h-1, where as the annual effective dose to the population was 0.09 mSv y-1. A correlation analysis was made between measured dose rate and individual radionuclides, in order to delineate the contribution of the respective nuclides towards dose rate. The radio-elemental concentrations of uranium, thorium and potassium estimated for the soils, in the study area, indicated the enrichment of uranium series nuclide. The results of the present study were subsequently compared with international and national recommended values.

## Introduction

An understanding of radionuclide distribution and radioactivity level in an ambient environment is important for assessing the radiation exposure to the population in a region. Two of the prominent sources of external radiation are the cosmic rays and terrestrial gamma rays. Natural radionuclides in soil generate a significant component of the background radiation exposure to the population.[1] Investigations on terrestrial natural radiation have received worldwide attention and led to extensive surveys in many countries like Spain, Turkey, Nigeria, Malaysia, and Botswana.[1–5] The absorbed dose rate in air depends mainly on the radionuclide concentration in the soil, as the largest component of the gamma radiation comes from the terrestrial radionuclides and the cosmic rays. There is a direct correlation between terrestrial gamma and radionuclide concentrations in soil as it contains small quantities of radioactive elements like U and Th along with their progenies.[6] Radioactivity in soil depends on its formation and transport processes that were inbuilt since soil formation.[7] According to Gascoyne,[8] chemical and biochemical interactions influence the distribution patterns of uranium, thorium and their decay products. Therefore, the measurement of natural radioactivity in soil is important to determine the changes in the natural background radiation. Jaduguda is situated at the central part of the Singhbhum shear zone (SSZ), which is endowed with a number of copper and uranium deposits associated with nickel, molybdenum, bismuth, tellurium, and selenium.[9–11] Uranium mineralization is present in most parts of this belt, but at few places it is significant in terms of economic values. The Jaduguda uranium deposit is the oldest one in the entire belt and has been mined from last four decades.[12] The natural background radiation of the study area has been investigated and reported by various authors.[13–16] The aim of the present study is to determine the radionuclide activity concentration in soil samples collected from different villages around Jaduguda, and to evaluate the annual effective dose from the ambient radioactivity.

### Geology of the study area

**Figure 1:**
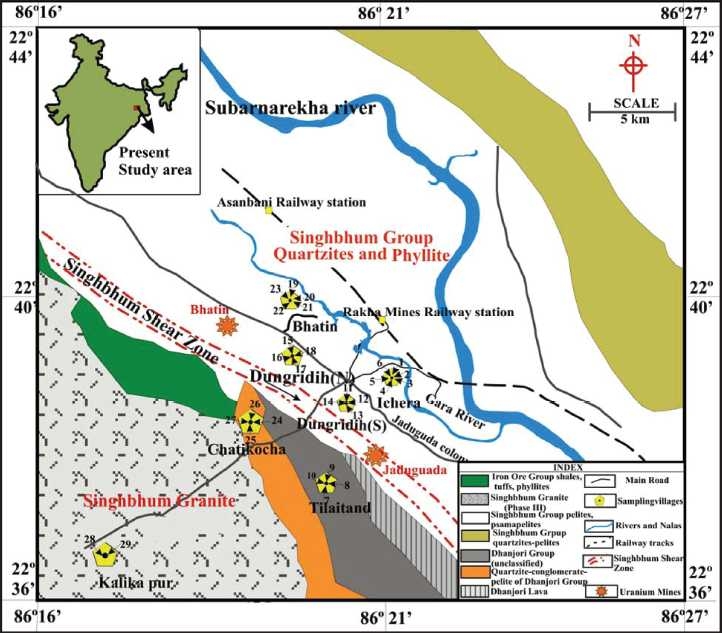
Geological map of Jaduguda along with the sampling villages

The simplified geological map of the study area is provided in [Figure 1] redrawn from the geological map provided by Sarkar.[17] The study area lies in Proterozoic metasediments of the SSZ in Jharkhand, eastern India.[18] Significant geological formations which host the radioactive minerals are provided in Figure 1. The SSZ includes the Singhbhum Group (SG) of rocks in the north, the Dhanjori Group (DG) of rocks, and Singhbhum Granite (SBG) in the south.[19] The SG consists of a monotonous rock sequence of pelitic schists, intercalated with micaceous quartzites, and mafic to ultramafic volacanics.[20,21] The DG consists of quartzite and metapelite in the lower part and mafic and ultramafic tuffs with low-Al, quartz normative tholeiitic lavas in the upper part.[9,19] SBG-III is granodiorite to granite composition.[22] The area also included rocks, which are mylonitized due to shearing in the SSZ. The major rock types are found in the study area includes apatite-tourmaline-magnetite-biotite-chlorite-quartz schist (Granular rock), brecciated quartzite, autoclastic conglomerate, and biotite chlorite schist, which host the radioactive minerals.[21] The present study covered seven villages namely, Ichera (22° 9′ 38.3″ N; 86° 21′ 47.5″ E), Tilaitand (22° 38′ 56.9″ N; 86° 20′ 20.07″ E), North Dungridih (22° 39′ 22.3″ N; 86° 20′ 35.9″ E), South Dungridih (22° 39′ 11.0″ N; 86° 20′ 26.2″ E), Chatikocha (22° 38′ 57.1″ N; 86° 20′ 06.1″ E), Bhatin (22° 40′ 10.5″ N; 86° 20′ 11.4″ E), and Kalikapur (22° 37′ 05.5″ N; 86° 17′ 29.2″ E) at a distance of about 1-10 km radius in the vicinity of the Jaduguda uranium mineralized zone. Surface soil samples were collected from 29 locations for gamma measurement. Bhatin and Ichera villages are overlying the SG formation comprising mica schists. North and South Dungridih villages are overlying the mylonitic rock formation, which is the signature of shearing. Chatikocha and Tilaitarn villages are overlying the DG of metasedimentary rocks and Kalikapur village is situated over the SBG.

## Materials and Methods

### Sample collection

The soil samples were collected from 29 selected locations [Figure 1] along the seven villages. The sampling locations were chosen on the basis of the measured gamma dose rate (*µ*R h^−1^) readings and taken from a height of about 1 m from the ground by a portable survey meter (type UR 705, www.nucleonix.com) which provided different gamma levels from different rock types underlying the villages. It is presumed that the soils were derived from the respective rock types in the villages studied. Undisturbed soil samples were collected from the same location where the survey meter readings were taken, following the standard techniques given by the EML[23] Procedure Manual.

#### Survey instrument

A portable micro-R Survey Meter, type- UR 705 (manufactured by Nucleonix Systems Private Limited, Hyderabad, Andhra Pradesh, India, info@nucleonix.com) was used for *in situ* gamma level measurements. It is designed around an integrally coupled 2.54 cm × 2.54 cm scintillator coupled to a 2.54 cm × 1.27 cm photomultiplier tube. The detector assembly is within the survey meter enclosure. It has a sensitivity of 1 *µ*R h^−1^ with accuracy better than ±15% with a ^137^Cs source and a dead time of 16s which changes automatically depending on the rate level. The detection of gamma rays of cosmic sources is negligible due to the low response of the instrument to high-energy (1-5 MeV) gamma radiations. It detected a reading of only about half a division on the smallest scale of 1 *µ*R h^−1^ (~8.7 nGy h^−1^). This detector is the most widely used device for all kinds of gamma ray surveys in India due to its efficiency.

### Sample preparation for γ-ray spectrometry analysis

The soil samples were dried in an oven at 110° C overnight, to remove moisture, pulverized, homogenized, and sieved through a 2 mm mesh. About 350 g of samples was filled and sealed in leak-proof, air-tight PVC containers of 6.5 cm diameter and 7.5 cm height, weighed and stored for a period of 1 month for secular equilibrium between radium and thorium with their daughter nuclides. Each sample was then analyzed by HPGe gamma ray spectrometry.

#### Gamma ray detection system

**Figure 2:**
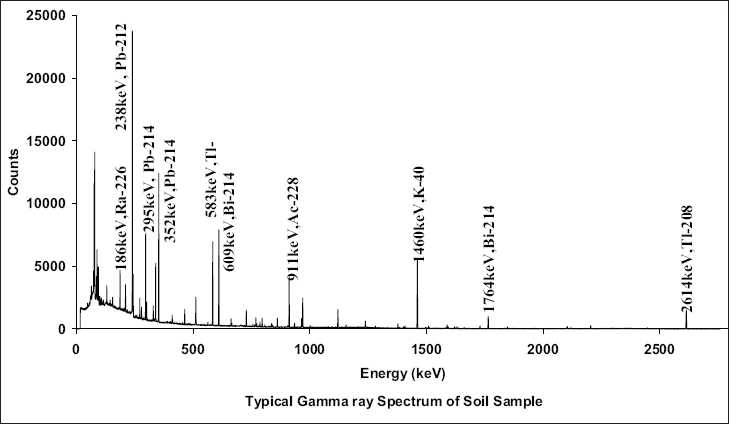
Typical gamma spectrum obtained from the HPGe spectrometer

The concentrations of ^238^U, ^232^Th, and ^40^K were determined by high-resolution gamma spectrometry. It is the most reliable method for the estimation of uranium, thorium, and potassium activity concentration which is widely used in India for geological point of view and has been used by various workers in this field.[24] The detector is a p-type HPGe detector with a relative efficiency of 50%. This facility is available at Radiation Safety Systems Division, BARC, Mumbai, India. The detector was covered with a 3” lead shield on all sides to reduce the background due to natural radioactivity. The resolution of the detector was 1.9 keV for 1332 keV of ^60^Co. The output of the detector system was connected to a PC-based 8k multichannel analyzer (Gamma Fast). The IAEA standard reference materials, uranium ore (RGU-1), thorium ore (RG Th), and KCl power of known activity, were used for the calibration of the system. The peak areas of the prominent gamma energy lines from ^234^Pa (766 and 1001 keV), ^226^Ra (186 keV), ^214^Pb (241, 295, 352 keV), and ^214^Bi (609, 934, 1120, 1238, 1377, 1764, 2204, and 2448 keV) isotopes were used for the efficiency calibration of the HPGe Gamma ray spectrometer. The efficiency for each gamma energy E is calculated and it is fitted in a semi-empirical mathematical equation given by Eq. (1):[25]

(1) $$\mathrm{Ln}\left(\mathrm{Eff}\right)\;=\;A\;+\;\mathrm{BLn}\left(E\right)\;+\;C{\left(\mathrm{Ln}\left(E\right)\right)}^{2}$$

where *A*, *B*, and *C* are fitted coefficients. Using the above function, the efficiency for different energies can be calculated. Each sample was counted for 50 ks to reduce the counting uncertainity. Assuming that the daughter products of uranium and thorium will be in equilibrium, gamma lines from the daughter products of ^226^Ra and ^228^Ra are used for the present analysis. For uranium estimation, the following gamma energy peaks were used: 352 keV from ^214^Pb, 609 keV and 1764 keV from ^214^Bi. Similarly for thorium estimation, 911 keV from ^228^Ac, 238 keV from ^212^Pb, 583 keV from ^208^Tl gamma peaks were used. The ^40^K activity was estimated using the 1460 keV gamma peak. The activity concentration was estimated from the intensity of each line taking into account the mass of the sample, the branching ratio of the gamma decay, the time of counting and the efficiencies of the detector. A gamma ray spectrum recorded with a HPGe detector is shown in Figure 2, with the gamma lines from various daughter nuclides of ^232^Th, ^238^U, and ^40^K from the soil samples.

The gamma absorbed dose rates in air at a height of 1 m above the ground surface was calculated from the activity concentration of ^238^U, ^232^Th, and ^40^K isotopes present in the soil. The conversion factor described by UNSCEAR[26] was adopted to estimate the absorbed dose rates using the equation given below:

(2) $$D\;=\;\left(0.604C_{\mathrm{Th}}\;+\;0.462C_{U}\;+\;0.0417C_{K}\right)\left({\mathrm{nGyh}}^{-1}\right)$$

where 0.604, 0.462, and 0.0417 are dose conversion factors (nGy h^−1^ per Bq kg^−1^) and C_Th_, C_U_, and C_K_ are the radionuclide concentrations for ^238^U, ^232^Th, and ^40^K, respectively. The effective dose received by adults was then estimated from the absorbed dose (nGy h^−1^) in air by using a conversion factor 0.7 (SvG y^−1^) and an occupancy factor (0.2) for outdoor environment as given by UNSCEAR[26] from the following equation:

(3) $$\mathrm{Effective}\;\mathrm{dose}\;\left(\mathrm{mSv}\;y^{-1}\right)\;=\;\mathrm{Dose}\;\mathrm{rate}\;\left(\mathrm{nGy}\;h^{-1}\right)\;\times \;24\;\left(h\right)\;\times \;365.25\;\left(d\right)\;\times \;0.2\;\times \;0.7\;\times \;10^{-6}$$

## Results and Discussions

### Radioactivity concentration levels

**Figure 3:**
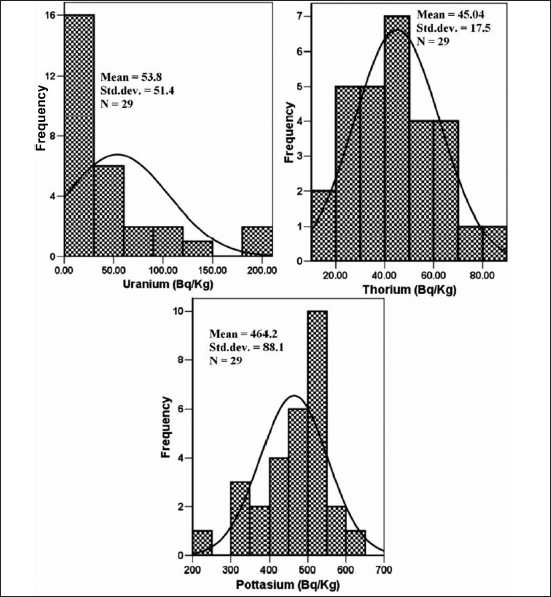
Frequency distribution of ^238^U, ^232^Th, and ^40^K concentration in the soil samples from Jaduguda

The radionuclide concentration for the collected surface soil samples indicates the variability of geological formation for the area studied. Table 1 summarizes the specific activity of ^238^U, ^232^Th, and ^40^K in the soils in Bq kg. The ^238^U activity is distinctly higher than the ^232^Th and it ranges from 20.1 to 120.0 Bq kg^−1^ with a mean activity of 53.8 ± 3 Bq kg^−1^. The ^232^Th concentration ranges from 23.8 to 69.9 Bq Kg^−1^ with a mean activity of 45.0 ± 2.1 Bq kg^−1^. The ^40^K concentration ranges from 396.5 to 513.8 Bq kg^−1^ with a mean activity of 462.2 Bq kg^−1^. The study area comes under the shear zone and contains a relatively higher concentration of uranium and less thorium, which reflects in the soil radioactivity concentration.[12] Soils are considered as the weathered products of rock types and the distribution of radioactive elements is immensely affected by processes like weathering and erosion. Soil radioactivity mainly depends on the types of rock from which the soil originates.[27] The highest concentration of ^238^U was found in North Dungridih and South Dungridih villages, whereas the ^232^Th and ^40^K concentration was found similar in all the villages. The difference in the specific radionuclide concentration in the villages may be related to the underlying bed rock types and local geology of the study area. A frequency distribution was attempted to show the distribution of radionuclides in the study area and is provided in Figure 3. The ^238^U activity fitted to the log normal distribution with an asymmetrical curve indicating its dominance in a particular region, whereas ^232^Th and ^40^K fitted to a normal distribution with symmetrical curves indicating an even distribution throughout the study area. The global average of ^238^U, ^232^Th, and ^40^K in soil is 35, 45, and 420 Bq kg^−1^, respectively.[26] In the Indian context, these values are 31, 63, and 394 Bq kg^−1^, respectively.[28] The results obtained from the present study were compared to the world as well as Indian average values shown in Table 2.

**Table 1:** Activity concentrations of ^238^U, ^232^Th, and ^40^K in soil samples around Jaduguda

*Region*	*Activity concentration (Bq kg^−1^)*		
	*Uranium (^238^U)*	*Thorium (^232^Th)*	*Potassium (^40^K)*
Ichera (6)	31.2 ± 2.5	50.1 ± 2.1	468.9 ± 38.6
Tilaitarn (4)	20.1 ± 2.4	23.8 ± 1.8	405.5 ± 25.7
North Dungridih (4)	118.5 ± 4.5	47.8 ± 2.1	513.8 ± 27.2
South Dungridih (4)	120.0 ± 4.3	69.9 ± 2.6	513.0 ± 29.6
Chatikocha (4)	40.7 ± 3.2	50.1 ± 2.2	441.9 ± 28.9
Bhatin (5)	21.8 ± 2.1	27.2 ± 1.9	396.5 ± 25.7
Kalikapur (2)	24.0 ± 2.0	40.5 ± 2.0	562.6 ± 31.7
Range	20.1±120.0	23.8±69.9	396.5–513.8
AM	53.8 ± 3	42.2 ± 2.1	471.7 ± 29.6
SD	45.2	15.6	61.4

± value indicates the error associated with the measurement, *n* indicates the number of samples from respective villages

**Table 2:** Comparison of the results obtained from soil samples of Jaduguda with other countries

*Sampling villages (n)*	*Activity concentration (Bq kg^−1^)*			Reference
	*^238^U*	*^232^Th*	*^40^K*	
Jaduguda (villages)	53.8	45.0	464.2	Present study
Jaduguda	50.67–109.14	28.73–89.78	989.84–1121.86	Chakrabarty *et al*.[16]
Roadside soil, Musabani–Jamshedpur road (India)	16.6–390.5	21.1–148.2	85.9–881.6	Sahoo *et al*.[13]
West coast of India	30.6	38.2	152.2	Karunakara *et al*.[32]
Spain	46	49	650	Moreno *et al*.[2]
Turkey	115	192	1207	Baykara and Doğru *et al*.[3]
China	112	71.5	672	Yang *et al*.[^33^]
Botswana	34.8	41.8	432.7	Murty *et al*.[1]
World average	35	45	420	UNSCEAR[25]

The absorbed dose rate (*D*, nGy h^−1^), a relevant quantity when considering radiation risk to humans, at a height of 1 m above the ground surface due to the concentrations of ^238^U, ^232^Th, and ^40^K in the soil is estimated using Eq. (2) for all the sampling locations and presented in Table 3. The absorbed dose rate estimated from soil for Indian sub-continent is about 69 nGy h^−1^[28] and the world average is 51 nGy h^−1^.[26] In the present survey the average outdoor gamma dose for soil ranged from 41.8 to 119.6 nGy h^−1^ with a mean value of 72.5 nGy h^−1^ which agrees well with the global average values. The annual effective dose in the environment to the population was estimated using Eq. (3). The conversion coefficient of 0.7 Sv Gy^−1^ and an occupancy factor of 0.2 are provided according to UNSCEAR reports. The conversion coefficient gives the equivalent human dose in (Sv y^−1^) from the absorbed dose rate in air (nGy h^−1^), while the occupancy factor gives the function of the time an individual is exposed to outdoor radiation. Considering that people in India on an average spend ~20% of their time outdoors, the annual effective dose rates were calculated. In the present study, the effective dose rate ranged between 0.05 and 0.15 mSv y^−1^ with a mean value of 0.09 mSv y^−1^, which is similar to 0.07 mSv y^−1^ given in UNSCEAR as the worldwide representative value.

**Table 3:** Air-absorbed dose rate and annual effective dose at the villages of Jaduguda

*Sampling villages*	*Absorbed dose rate (nGy h^−1^)*	*Annual effective dose (mSv a^−1^)*	*External hazard index (H_ex_)*
Ichera	66.6	0.08	0.37
Tilaitarn	41.8	0.05	0.23
North Dungridih	105.2	0.13	0.61
South Dungridih	119.6	0.15	0.70
Chatikocha	69.5	0.08	0.40
Bhatin	43.1	0.05	0.25
Kalikapur	59.1	0.07	0.34
Range	41.8–119.6	0.05–0.15	0.23–0.70
Average	72.1	0.09	0.41
SD	29.7	0.03	0.17

### Correlation studies between the activity concentration and the measured dose rate

**Figure 4:**
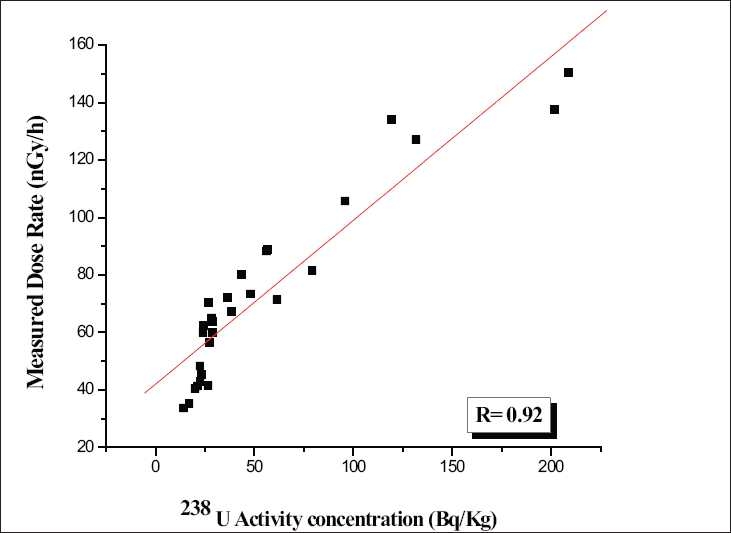
Scatter plot of the ^238^U versus measured dose rate

**Figure 5:**
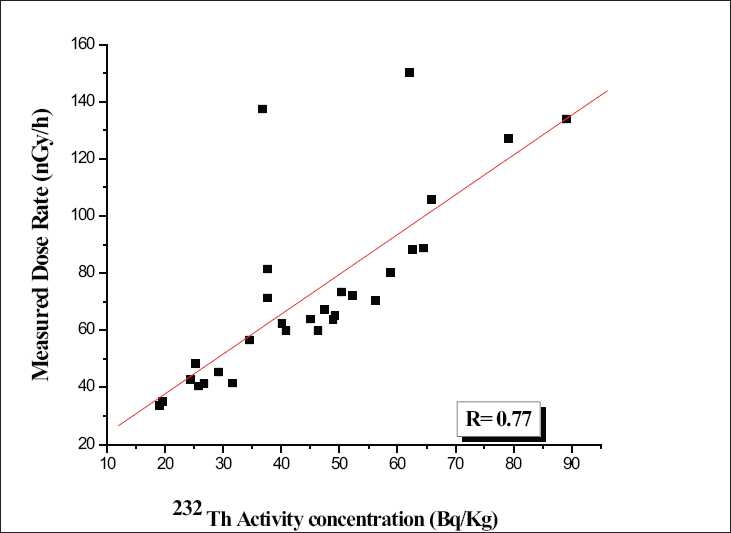
Scatter plot of the ^232^Th versus measured dose rate

**Figure 6:**
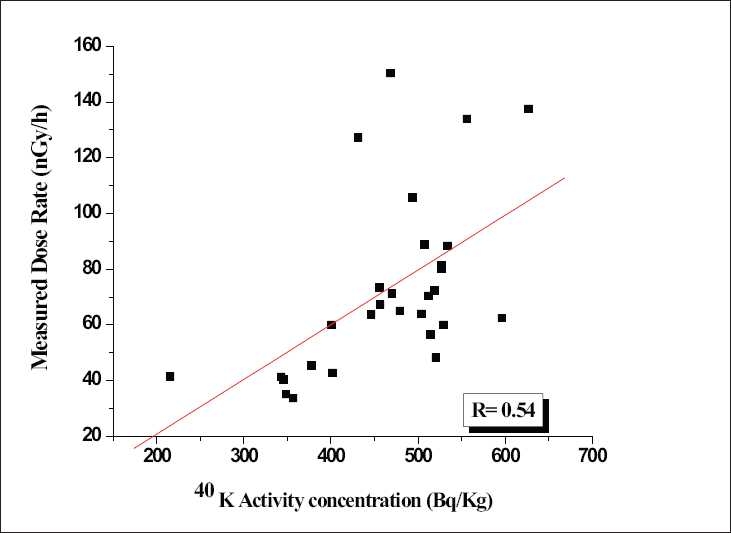
Scatter plot of the ^40^K versus measured dose rate

In order to find the existence of these radioactive nuclides together at a particular place, correlation studies were performed between combinations of radionuclides like ^238^U and ^232^Th and ^238^U and ^40^K. A very poor correlation observed between individual activity concentrations of radionuclides, which indicate individual results for any one of the radionuclide, is not a good predictor of the concentration of the other. Therefore, an attempt was made to delineate the contribution of individual radionuclides toward the measured gamma dose rate in the study area irrespective of the cosmic radiation. Correlation studies were performed between the measured dose rates with the respective radionuclide activity concentration. The correlation has been given in Figures 4–6. There is a good correlation between the ^238^U activity (0.92) and the measured dose rate as shown in Figure 4. A poor correlation existed between ^232^Th (0.77) and ^40^K (0.54) with the measured dose rate as shown in Figures 5 and 6. Since the study area is one of the major uranium deposits found in India, major portion of the dose may be contributed due to the ^238^U series as well as by ^232^Th series and a negligible activity due to ^40^K. The present ^238^U, ^232^Th, and ^40^K activities in the surface soils are due to both natural and anthropogenic activities in the study area.

### Estimation of external hazard indices

The decay of naturally occurring radionuclides (^238^U, ^232^Th, and ^40^K) in soil produces a radiation field that also crosses the soil–air interface to produce significant human exposure. The external hazard index, *H*_ex_, is calculated and examined according to the following equation:[30]

(4) $$H_{\mathrm{ex}}\;=\;C_{U}/370\;+\;C_{\mathrm{Th}}/259\;+\;C_{K}/4810\;\leq \;1$$

The *H*_ex_ value must be less than 1 to keep the radiation hazard insignificant to the people. The calculated *H*_ex_ value for the soil samples studied ranged between 0.23 and 0.70 with an average value of 0.41 [Table 2]. These values are far below the criterion limit as per European Commission of Radiation Protection reports.[31] The surface soil from the villages has no high exposure for either inhabitants and can be used as a construction material without posing any significant remediation.

### Gamma dose rate Isodose map

**Figure 7:**
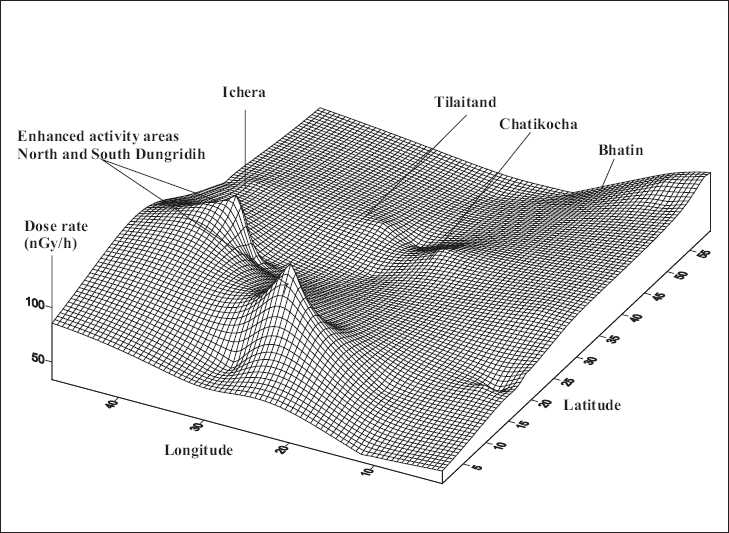
The 3D plots of Isodose map of Jaduguda showing the sampling villages

The terrestrial gamma dose rates at each sampling village are plotted using SURFER (software version 8). Figure 7 shows the 3D plot of the computer-generated gamma Isodose map of the region studied. The enhanced activity areas are indicated with higher peak regions in the plotted map.

## Conclusions

The results obtained from the present work provide additional data on the radioactivity levels in the surface soil from the villages around Jaduguda. The measured activity concentrations of radionuclides and gamma dose rates are compared with the global and Indian average values. Gamma spectrometric analysis of the soil samples indicates that the activities due to major radionuclides in the uranium series are significantly high as compared to thorium and potassium. This is due to the widespread uranium mineralization in the study area. There is a good correlation between ^238^U series with the gamma dose rate. However, it is observed that in two villages, i.e., North and South Dungridih, the activity concentration of ^238^U is higher as compared to the other villages studied. The value of the external hazard index determined in the soil is less than the recommended levels.
